# Atrial Fibrillation and Obstructive Sleep Apnea: Do Mortality Trends Reflect Disease Burden or Diagnostic Gaps?

**DOI:** 10.1002/clc.70200

**Published:** 2025-08-28

**Authors:** Naoya Kataoka, Teruhiko Imamura

**Affiliations:** ^1^ Second Department of Internal Medicine University of Toyama Toyama Japan

**Keywords:** anticoagulation, catheter ablation, heart failure, stroke

To Editor,

The authors demonstrated a striking and consistent rise in atrial fibrillation (AF)–related mortality involving obstructive sleep apnea (OSA), particularly among elderly, female, rural, and minority populations [1]. This important analysis highlights the growing public health burden of AF–OSA overlap. However, several issues merit further clarification and discussion.

The authors report a steep increase in AF‐related mortality involving OSA over the past two decades [1]. However, as AF prevalence and mortality have also generally increased in the U.S. population [2], it is unclear whether the observed trend is specific to patients with comorbid OSA or merely reflects overall AF epidemiology. A comparative analysis of mortality trends in AF patients without OSA would be helpful to determine the additive risk associated with OSA.

During the study period (1999–2020), catheter ablation became increasingly adopted for rhythm control in AF [3]. Recent studies suggest that AF ablation is associated with improved long‐term outcomes, particularly in younger male patients [4]. Yet, despite these advances, AF‐related mortality in patients with OSA continued to rise. How do the authors interpret this apparent contradiction? Were these procedures less commonly used or less effective in the OSA subgroup?

The study identifies AF (ICD‐10 I48.x) as the “underlying” cause of death and OSA (G47.33) as a “contributing” condition [1]. However, the clinical circumstances in which AF is recorded as the primary cause of death—distinct from its role as a comorbidity in stroke, heart failure, or sudden death—are not well defined. It would be informative to clarify how “AF‐related mortality” was operationalized in this study and whether misclassification or variation in coding practices may have influenced the observed trends.

The authors correctly point out the rising burden of AF‐related mortality in the presence of OSA [1]. Yet over the same period, mortality from stroke and heart failure—two major downstream complications of AF—has generally declined, partly due to improvements in anticoagulation and HF management [5]. This discrepancy raises the question of whether the increase in AF‐related deaths reflects actual clinical deterioration or improved documentation of AF on death certificates.

While the use of CDC WONDER provides valuable national‐level insights [1], the reliance on death certificate data introduces several limitations. OSA is often underdiagnosed, particularly among women, minorities, and older adults—groups in whom mortality increases were most pronounced. Additionally, data on OSA severity, AF subtype, comorbidities (e.g., heart failure, chronic kidney disease), and continuous positive airway pressure adherence were not available. These unmeasured variables may have played an important role in shaping the observed trends.

## Conflicts of Interest

The authors declare no conflicts of interest.

## Data Availability

The authors have nothing to report.

## References

[1] I. N. Hassan , M. Ibrahim , S. Yaqub , et al., “Trends in Atrial Fibrillation‐Related Mortality Among Older Adults With Obstructive Sleep Apnea in the United States, 1999–2020,” Clinical Cardiology 48 (2025): e70178. 40662442 10.1002/clc.70178 PMC12261039

[2] J. Wu , R. Nadarajah , Y. M. Nakao , et al., “Temporal Trends and Patterns in Atrial Fibrillation Incidence: A Population‐Based Study of 3.4 Million Individuals,” Lancet Regional Health–Europe 17 (2022): 100386. 35721699 10.1016/j.lanepe.2022.100386 PMC9198843

[3] A. Lappalainen , J. E. K. Hartikainen , K. Teppo , et al., “Temporal Trends of Catheter Ablation Procedures in Patients With Atrial Fibrillation and Atrial Flutter: A Nationwide Cohort Study,” IJC Heart & Vasculature 55 (2024): 101541. 39507295 10.1016/j.ijcha.2024.101541 PMC11539521

[4] Z. U. A. Asad , A. Yousif , M. S. Khan , S. M. Al‐Khatib , and S. Stavrakis , “Catheter Ablation Versus Medical Therapy for Atrial Fibrillation: A Systematic Review and Meta‐Analysis of Randomized Controlled Trials,” Circulation: Arrhythmia and Electrophysiology 12 (2019): e007414. 31431051 10.1161/CIRCEP.119.007414

[5] M. Ding , M. Ebeling , L. Ziegler , A. Wennberg , and K. Modig , “Time Trends in Atrial Fibrillation‐Related Stroke During 2001–2020 in Sweden: A Nationwide, Observational Study,” Lancet Regional Health—Europe 28 (2023): 100596. 37180742 10.1016/j.lanepe.2023.100596 PMC10173271

